# How to make your research jump off the page: Co-creation to broaden public engagement in medical research

**DOI:** 10.1371/journal.pmed.1003246

**Published:** 2020-09-14

**Authors:** Nina Finley, Talia H. Swartz, Kevin Cao, Joseph D. Tucker

**Affiliations:** 1 Faculty of Infectious and Tropical Diseases, London School of Hygiene and Tropical Medicine, London, United Kingdom; 2 Division of Infectious Diseases, Icahn School of Medicine at Mount Sinai, New York, New York, United States of America; 3 Institute for Global Health and Infectious Diseases, School of Medicine, University of North Carolina at Chapel Hill, Chapel Hill, North Carolina, United States of America; 4 Social Entrepreneurship to Spur Health (SESH) Global, Guangzhou, China

## Abstract

Nina Finley and co-authors discuss public involvement in planning and reporting medical research.

**Summary points**

- Many scientific research manuscripts are intended for other researchers and not the public. However, the public are involved in research as participants, taxpayers, and patients.
- We discuss co-creation and how it can be used to enhance medical research.
- Co-creation is an iterative, bidirectional collaboration between researchers and laypeople to create knowledge. This process can broaden public engagement in medical research.
- Co-creation is related to theories of crowdsourcing, community-based participatory research, citizen science, and participatory action research.
- Public online calls for input, crowdsourcing contests, hackathons, and participatory design sessions are all examples of activities to co-create with the public.
- Infographics and videos are two tools that can be used to broaden public engagement in medical research.

How can you make your medical research jump off the page? Every year, 1.7 million peer-reviewed manuscripts are published, and many are never cited or shared [1]. These manuscripts are written by researchers for researchers. Paywalls, dense text, few illustrations, and complicated statistics prevent most of the public from seeing the end products of medical research. Yet the results of biomedical research are meant for the public. People living with diseases and other members of the public are often the ones who join (i.e., as research participants), fund (i.e., as taxpayers), and benefit from (i.e., as patients) medical studies. We propose using co-creation to broaden public engagement on medical research. Co-creation is an iterative, bidirectional collaboration between researchers and laypeople to create knowledge [2]. Public engagement is a mutually beneficial interaction between specialists and nonspecialists [3]. In medical research, public engagement occurs when a layperson reads, understands, and shares a publication. Public engagement could increase the number of people who read and understand medical research publications.

Public engagement benefits many groups. For researchers, public engagement can improve research quality [4], consolidate external support, enhance dissemination of results, expand readership, and boost impact [5]. For laypeople, engagement provides an opportunity to contribute to and learn about processes that affect their health. It gives patients more voice [6] and power [4] and holds researchers accountable to funders and beneficiaries. When done well, public engagement builds trust between researchers and the public. At its best, public engagement can spur systemic change in policy or practice.

## Methods

We identified systematic reviews, randomized controlled trials, and observational studies that show how co-creation can be used to enhance public engagement in medical research. We searched three databases using the terms “co-creation,” “public engagement,” and “research manuscript.” The search was initially undertaken January 20, 2019, and updated June 19, 2020. Although we included some theoretical literature related to co-creation, the focus was on applications related to writing research manuscripts. In this narrative opinion piece, we introduce the conventional approach to public engagement and suggest co-creation as a tool to help medical researchers engage the public. As part of the piece, we issued a public online call to solicit feedback on an infographic on June 18, 2019 [7]. An infographic is an image that presents information in a manner easily understood by nonexperts. The open call noted that suggestions would be used to improve the infographic and that compiled open access resources would be shared.

## The problem

The conventional approach to public engagement in medical research is one of benign neglect. A systematic review found that patient engagement was feasible in many medical research settings [8]. However, public engagement has generally been limited to the early phases of a study and not the final phases of creating a manuscript [8]. For example, engagement in clinical trials often takes the form of a community advisory board reviewing ways to optimize participant recruitment from the perspective of people living with the disease. While this input is useful for developing studies, it risks lapsing into tokenistic relationships between researchers and community members [8]. Fewer research studies engage the public in later research phases, such as developing a manuscript and sharing findings in a way that could be understood by the public.

## Co-creation with the public

Co-creation is an iterative, bidirectional collaboration between researchers and laypeople to create knowledge. We focus on co-creation as it relates to writing medical research manuscripts. Co-creation could include making research results available to the public earlier, in the form of a preprint or other publicly accessible form. Co-creation provides a structured process for broadening public engagement in research. This process is related to several types of engagement models, including crowdsourcing [9], citizen science [10], community-based participatory research [11], youth participatory action research [12], and patient and public involvement [13]. Using co-creation in research introduces several questions about who should be involved, extent of participation, acknowledging and recognizing participation, and related ethical issues [14, 15]. Co-creation approaches include public online calls for input, crowdsourcing contests [15], hackathons [16], and participatory design sessions [17].

**Fig 1:**
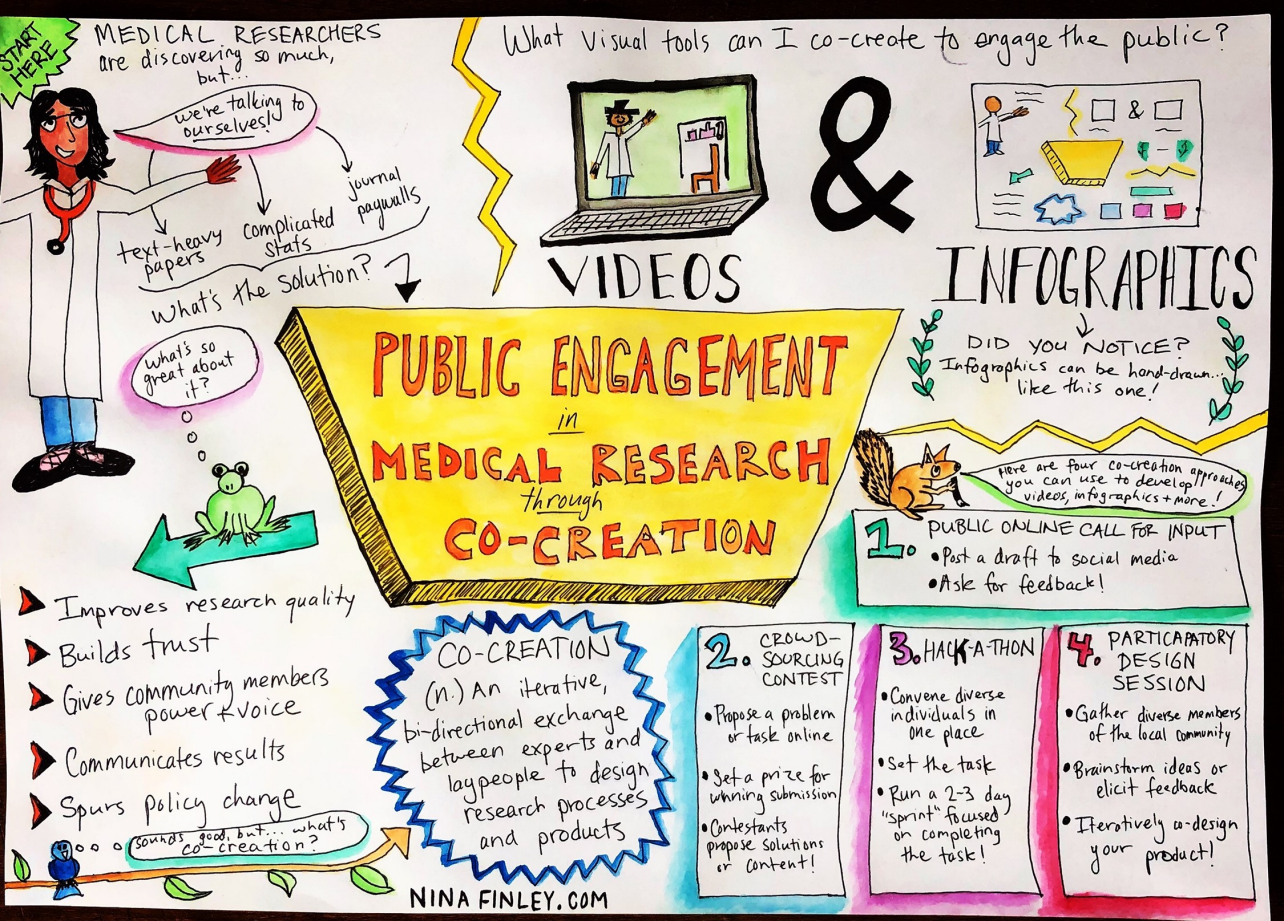
Co-creation in public engagement, developed using an open call through social media. Two authors posted the image on their respective Twitter feeds.

Public online calls for input are the simplest co-creation method. Social media platforms such as Facebook and Twitter allow researchers to post drafts of research content (e.g., infographics, preprints, videos) and receive real-time feedback from experts and nonexperts alike. For example, in creating this manuscript, we posted a draft infographic online (Fig 1) in order to solicit public feedback. The message resulted in 2,647 impressions (the number of times a tweet shows up in someone’s timeline) according to Twitter analytics, resulting in helpful feedback that improved the message (Fig 1). In addition, preprints allow the public free access to scientific research.

Crowdsourcing contests (also called innovation challenges, challenge contests, and prize contests) allow a group of individuals to tackle a problem and proposes solutions [9]. Contests have been used to create concepts, images, videos, and songs related to medical research. A recent systematic review identified 188 studies that used crowdsourcing in health and medical research [18]. The steps of conducting a crowdsourcing contest are typically to identify a steering group, solicit ideas from the public online, and select exceptional solutions to use or publish [15]. For example, a Chinese public health group used crowdsourcing to develop a campaign focused on increasing rates of testing for HIV (S1 Fig) [4]. This project had members of the public designing messages, images, and service models that were ultimately implemented in eight cities. Data from randomized controlled trials suggests that crowdsourcing contests are effective in creating sexual health messages [4, 19–21].

Hackathons (also known as hackfests, hack days, or designathons) are brief, sprint-like events in which individuals physically convene to focus on one topic for a short period [16]. Participants, judges, and steering committee members are often members of the public, and expert mentors are available to provide guidance. Although originally focused on developing computer software or hardware, hackathons are now used to spur innovation in both the content [22] and presentation [23] of medical research.

Participatory design sessions are in-person community gatherings organized by researchers. They can be used to brainstorm ideas, understand local perceptions of research, and elicit feedback on how results are presented. A team from Columbia University found that health-related infographics co-created with community members of diverse ages, languages, and health literacy levels were more informative, contextualized, and understandable for readers [17].

## Co-creating infographics and videos

Two excellent tools for public engagement are infographics and videos [6, 24]. These complementary tools are commonly accepted at major medical journals and may be useful for engaging public audiences. Infographics are similar to journal figures in that they are compact, data-rich visuals. The difference is that infographics should be easier to read and focus on one key message [25] and can engage people with varying literacy capabilities [17, 26, 27]. Infographics can be created by hand, as in the technique of sketch-noting, or by computer software. The key questions that need to be considered when creating an infographic are the “who, what, why, when, how, and where” of the message (Table 1). The University of Leeds and Public Health England published an open-access guide to creating infographics (S1 Text). This guide provides clear, user-tested advice on how to define the audience, align key components, and arrange visual elements. Infographics can be disseminated in many places: social media, email newsletters, blogs, or the local university bulletin board. A study in Northern Ireland found that patients who viewed an infographic were more likely to understand cancer risk factors than those who read the same information as text [26]. Two small studies suggest that people are more likely to read the abstracts of research articles with infographics than of those without [28, 29]. One Croatian study found that readers of Cochrane systematic reviews interpreted infographic and text summaries with equal accuracy, but enjoyed the infographics more [30].

**Table 1:** Key questions to answer in preparing an infographic or video related to medical research.

Who?	Who is the intended audience of this infographic/video and what are their visual preferences?
What?	What is the key message that needs to be conveyed? What can be simplified and what needs to retain complexity?
Why?	Why should the viewer care about this medical research finding or topic?
When?	What makes this topic urgent now? Why is now an important time to convey this infographic or video?
How?	How will this message be delivered to viewers (e.g., print, social media)?
Where?	Where is the group or groups that you intend to reach (e.g., geographic region, demographic group)?

Videos have the advantage of reaching some audiences who may not read medical research journals, including individuals for whom English is a second language and those who face socioeconomic barriers [6, 31, 32]. Short videos co-created with the public suggest new ways to empower groups that are underrepresented in research [24, 31, 33]. Video formats include whiteboard time lapses, filmed interviews with investigators, and short animations. Creating a video to explain key research findings does not require specialist training or resources of a professional production, such as a TED Talk (S2 Text). We have developed a guide for how to make a time-lapse video using only a whiteboard, smartphone, and basic video-editing software available on most computers (S3 Text). Researchers in China used a crowdsourcing contest to co-create sexual health videos with the public [20, 34]. The results showed that crowdsourced videos worked equally well or better than videos produced by a commercial social media firm. An example of a co-created video is included (S4 Text).

Co-creation to enhance public engagement has some limitations that should be noted. Sharing of potentially identifiable research data online must adhere to the same guidelines for ensuring patient confidentiality that exist with any identifiable research data. In instances in which identifiable information is shared, specific consent for online sharing is important. Second, infographics and videos will not replace conventional figures and tables in medical research articles. However, they could be a useful adjunct to extend public engagement. Third, there may be disciplines or settings in which the public may not be able or willing to be engaged. For example, research on proprietary materials or potentially traumatic topics (e.g., child maltreatment, female genital mutilation) may be less suitable for public engagement [35].

We encourage researchers to think beyond academic audiences and co-create with the public. What projects are you working on now that could lend themselves to co-creation? Start simple: try sketching an infographic of the results from your latest project and posting it on social media for public feedback. Craft a visual abstract when you submit your next research manuscript. Present your preliminary results to local community partners and incorporate their insights and wisdom; you may be surprised at how much your team and the local partners can gain from this process. The co-creation process can ultimately create greater transparency and accountability in research. Co-creation of research and visual aids can be the difference between a dusty manuscript on the shelf and an article that sparks conversation.

## Supporting information

S1 Fig Two images developed through a crowdsourcing contest. The contest had a steering committee, open call for submissions, evaluation of submissions, prizes awarded to finalists, and recognition of all those who contributed.
(DOCX)

S1 Text Open access resources for designing infographics for public health (noncommercial). (DOCX)

S2 Text Open access resources for designing videos. (DOCX)

S3 Text How to make a time-lapse video. (DOCX)

S4 Text Example of a co-created video presentation. (DOCX)

## Abbreviation

SESH: Social Entrepreneurship to Spur Health
