# Supplementary material for: How to make your research jump off the page: Co-creation to broaden public engagement in medical research
Source: PLoS Med. 2020 Sep 14;17(9):e1003246. doi: 10.1371/journal.pmed.1003246 (PMC7489547; doi:10.1371/journal.pmed.1003246)

**S1 Fig. Two images developed through a crowdsourcing contest. The contest had a steering committee, open call for submissions, evaluation of submissions, prizes awarded to finalists, and recognition of all those who contributed.**


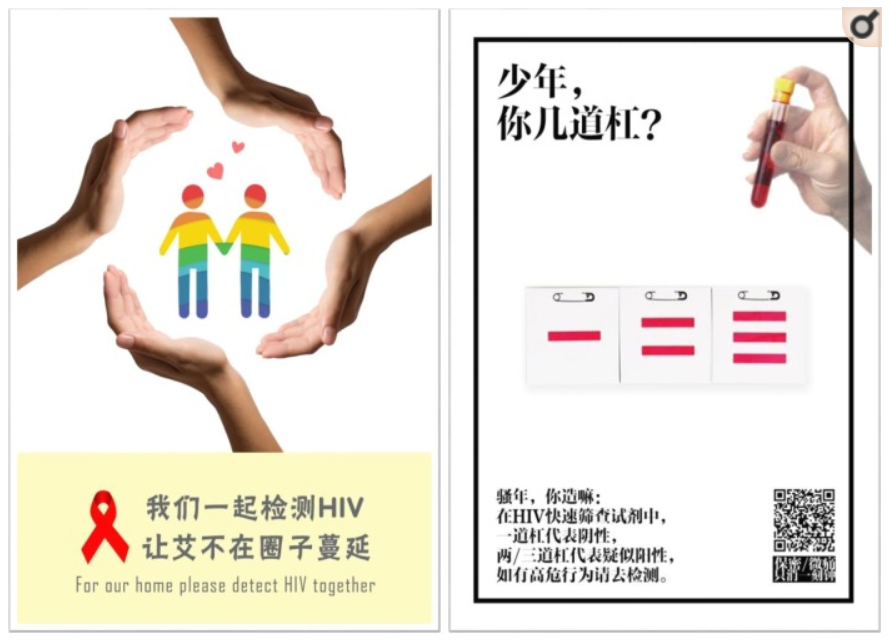

Supplement: S1 Fig — The contest had a steering committee, open call for submissions, evaluation of submissions, prizes awarded to finalists, and recognition of all those who contributed. (DOCX) [file pmed.1003246.s001.docx]
