# Supplementary material for: How to make your research jump off the page: Co-creation to broaden public engagement in medical research
Source: PLoS Med. 2020 Sep 14;17(9):e1003246. doi: 10.1371/journal.pmed.1003246 (PMC7489547; doi:10.1371/journal.pmed.1003246)
Supplement: S1 Text — (DOCX) [file pmed.1003246.s002.docx]

**S1 Text. Open access resource for designing infographics for public health (non-commercial).**

1. Visualising Health: https://visualisinghealth.files.wordpress.com/2014/12/guidelines.pdf
