# Supplementary material for: How to make your research jump off the page: Co-creation to broaden public engagement in medical research
Source: PLoS Med. 2020 Sep 14;17(9):e1003246. doi: 10.1371/journal.pmed.1003246 (PMC7489547; doi:10.1371/journal.pmed.1003246)
Supplement: S2 Text — (DOCX) [file pmed.1003246.s003.docx]

**S2 Text. Open access resources for designing videos.**

1. Video Production Tips (non-commercial from PennState): <https://mediacommons.psu.edu/2017/02/01/video-production-tips/>
2. Creating video abstracts (from BMJ Author Hub): <https://authors.bmj.com/writing-and-formatting/video-abstracts/>
