# Supplementary material for: How to make your research jump off the page: Co-creation to broaden public engagement in medical research
Source: PLoS Med. 2020 Sep 14;17(9):e1003246. doi: 10.1371/journal.pmed.1003246 (PMC7489547; doi:10.1371/journal.pmed.1003246)
Supplement: S3 Text — (DOCX) [file pmed.1003246.s004.docx]

**S3 Text: How to make a time-lapse video**

**Step 1: Gather materials**

You do not need professional equipment or experience to make a compelling time-lapse video. Most of the necessary materials can be found in your house, smartphone, and computer. You will need the following materials:

- pencil and paper to sketch out draft storyboard (a series of key visual elements) and video script
- dry erase whiteboard
- dry erase markers
- video recording device (most smart phones will work fine) to record video and audio
- basic video editing software (e.g., iMovie on Macs, Video Editor in Microsoft)
- an elevated surface (see one set-up below, although others have used tripods and large desks with compartments)


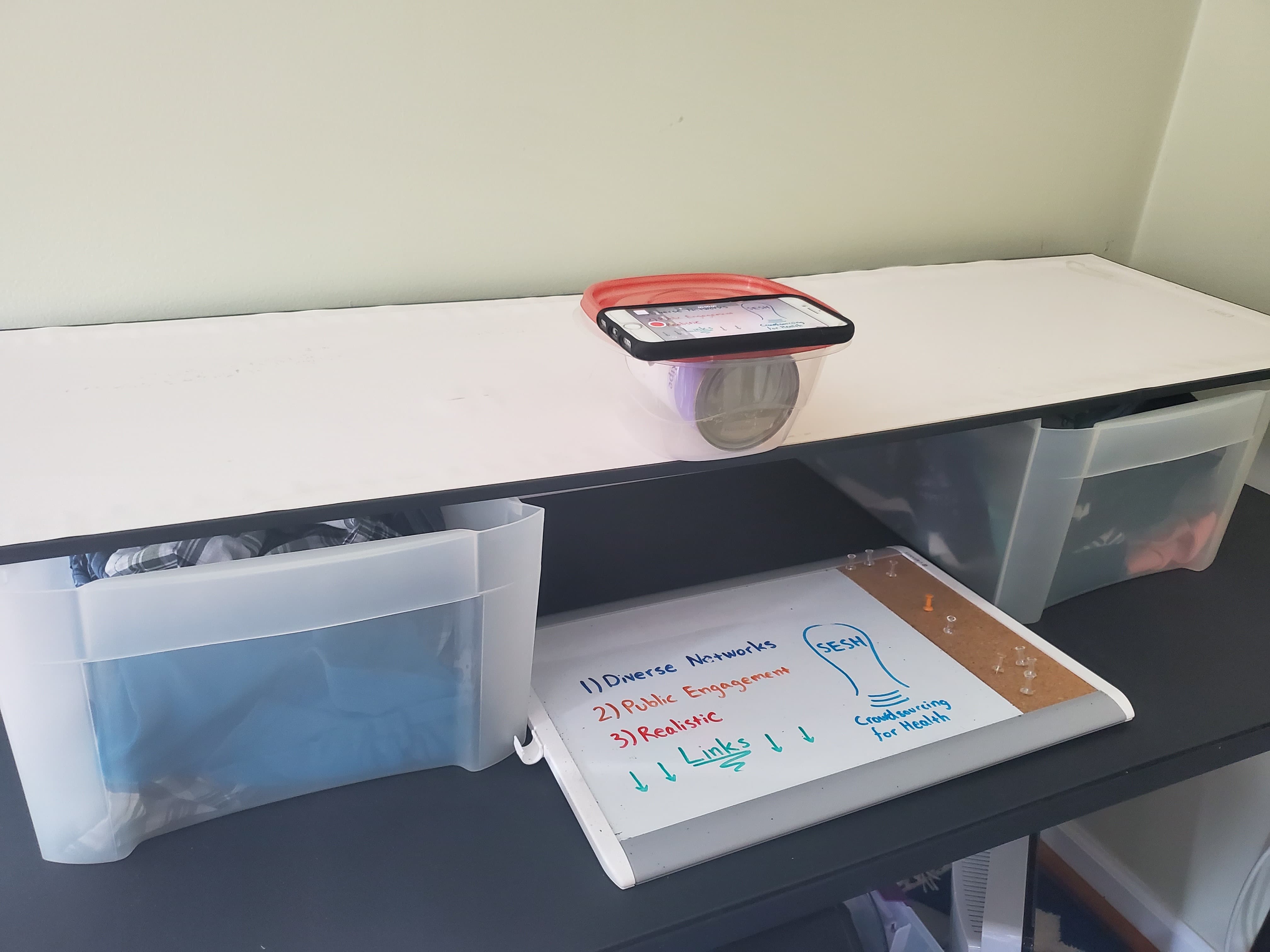


**Step 2: Create a storyboard and script for the audio**

Creating a storyboard and accompanying audio script can often take the most time. A storyboard is composed of several slides that are independent ideas. The drawing process is iterative and often goes through several stages. Sketch out the key visual elements of your research in a series of slides. What are the important parts that you would like your audience to understand? Will these pictures and concepts be understandable to a public audience? Avoid jargon and acronyms. Be concise. The drawings should be colorful, but not excessively so. Show your draft storyboard to a friend to gauge if they can understand the general idea. Alongside the storyboard, create an audio script that corresponds to each separate image.


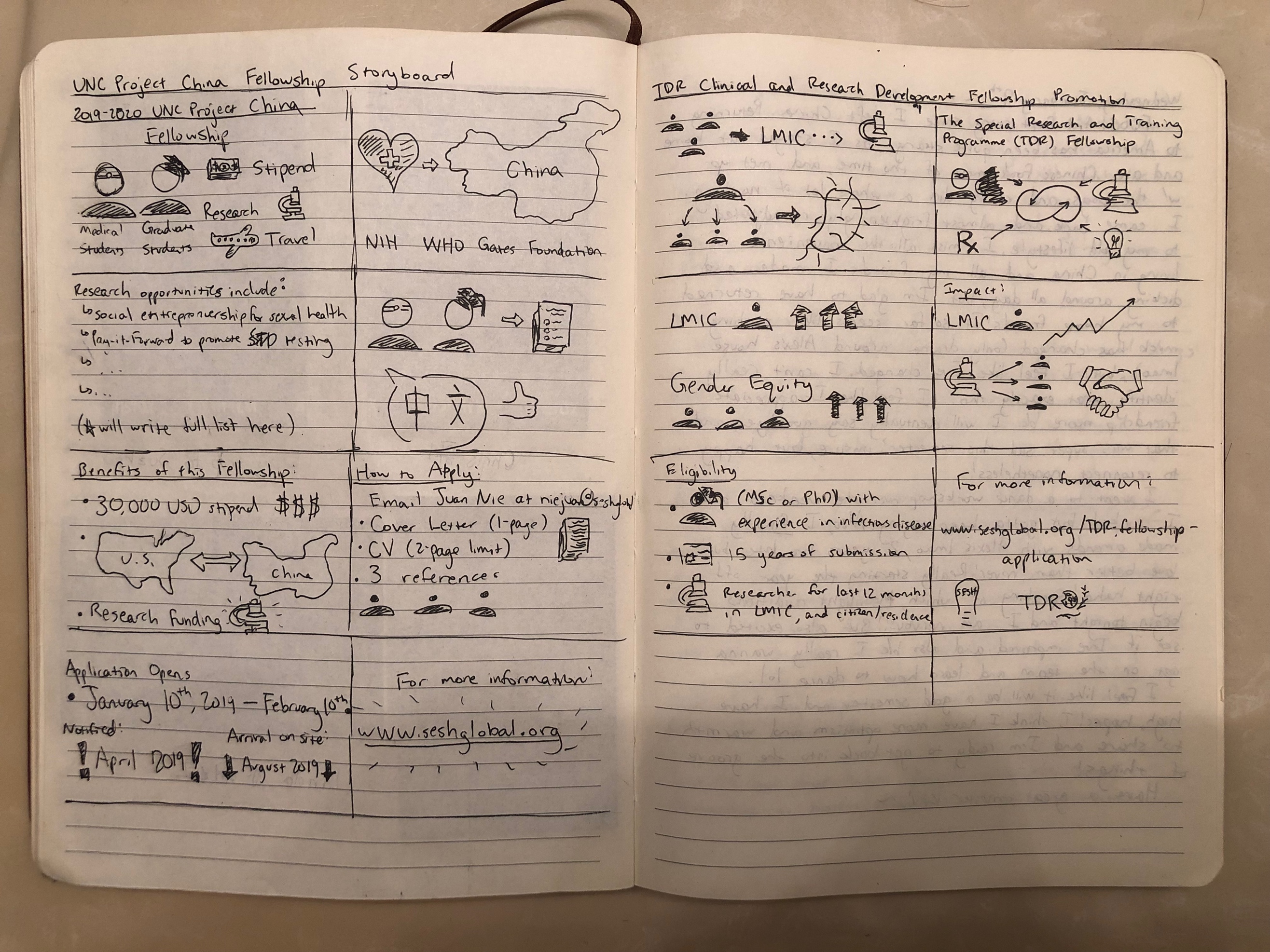


**Step 3: Prepare the recording set-up**

The next step is to set up the recording environment – no need for quiet since you will record your audio separately. You need to set up the smartphone so that it is resting on an elevated surface. The surface must be high enough so that the smartphone can capture a large enough part of the whiteboard, and it must be stable enough that it will not move or shake while drawing or erasing on the whiteboard. In this picture, I pulled two plastic drawer bins, stacked a mirror (to act as a stable, flat surface), placed a small box on top, and rested my smartphone on the edge so that the video camera is peeking over.


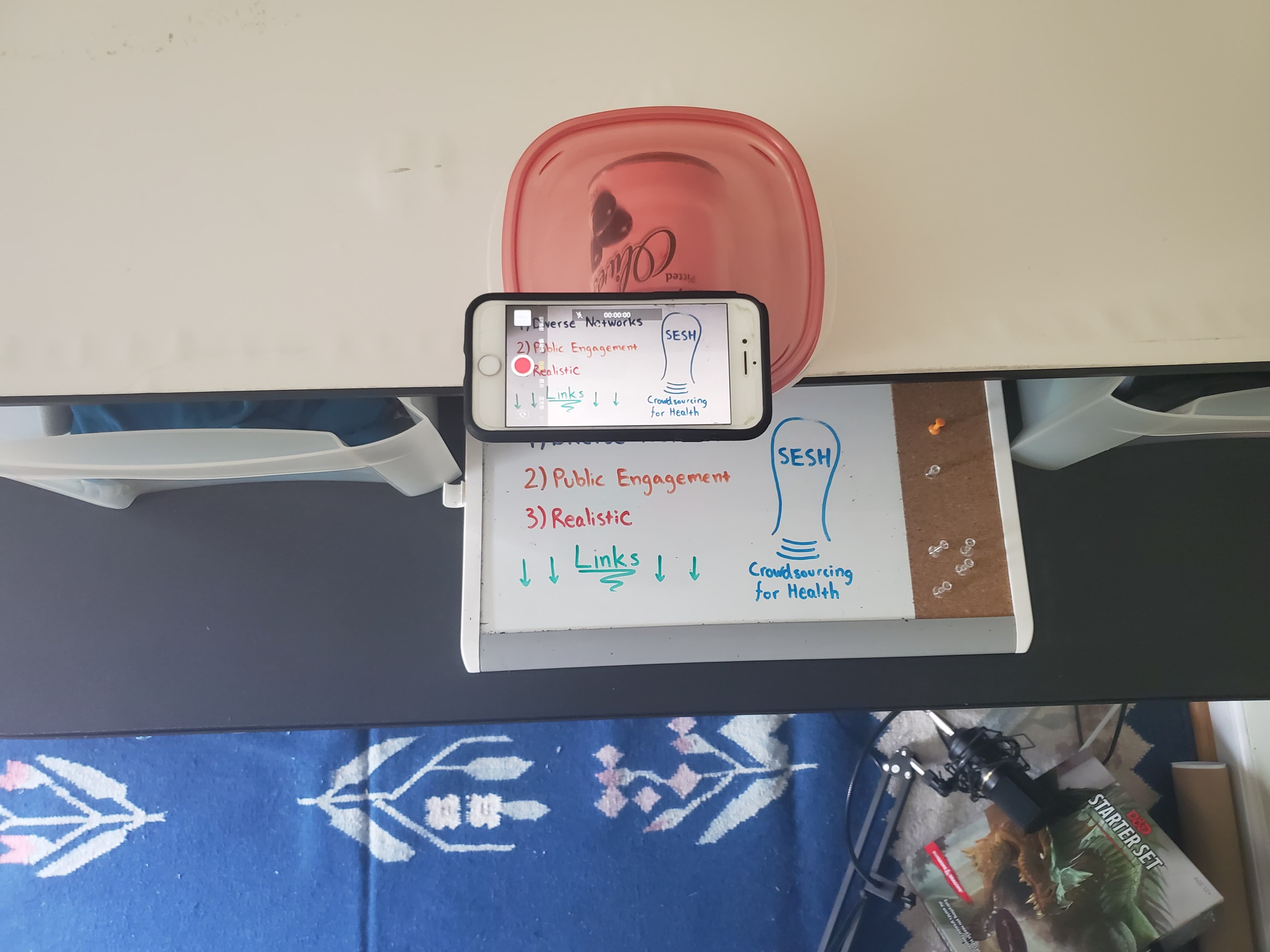


**Step 4: Record an initial drawing**

Once the recording set-up is finished, then you can move onto the drawing itself. First, draw a rough draft version of the video. This draft can be quickly drawn and edited to give a better sense of what the final video will look like and will help catch any last-minute changes that should be added to the final draft. Once the draft is reviewed and looks good, then one can move on to recording and editing the final draft.

**Step 5: Record the final drawing**

Be sure to pause briefly after completing each slide, so that editing the video will be made simpler. Once a slide is finished, then one can erase the board and begin drawing the following slide. Repeat until all slides have finished. Record audio based on the audio script.

**Step 6: Edit the video**

Import the video footage and script voiceover into the video editing software. Most simple video editing software should be able to speed up, slow down, and freeze frame video footage. This is what creates the time-lapse effect. Speed up the video so that it matches up with the script voiceover. Add freeze frames and adjust the amount that each clip is sped up so that the video matches up with the script voiceover.


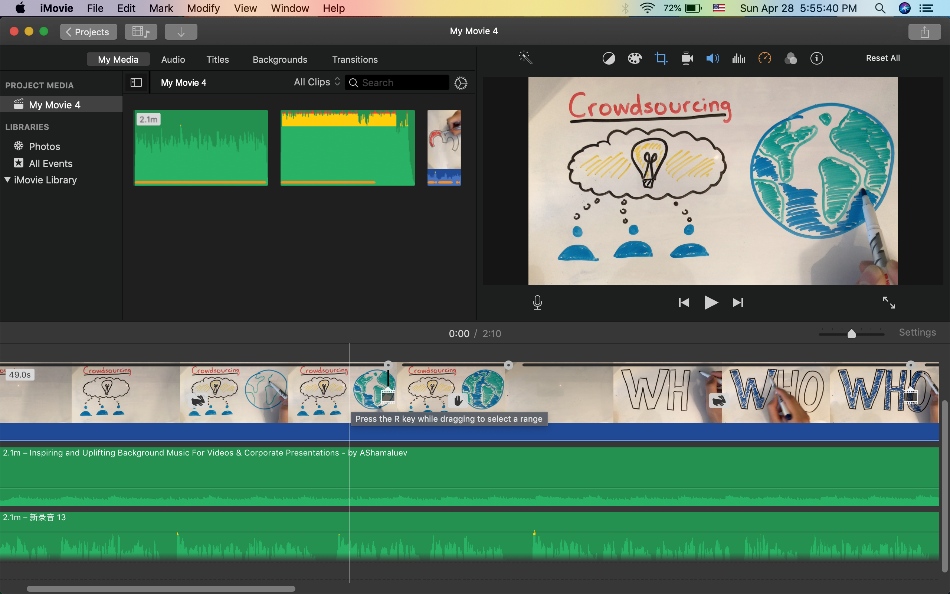


**Step 7: Add background music**

Consider adding background music which can generate excitement. There are many places to download free background music, simply search for “free business presentation background music” on Google or YouTube and download open access music that fits the goal of the video.
