# Supplementary material for: How to make your research jump off the page: Co-creation to broaden public engagement in medical research
Source: PLoS Med. 2020 Sep 14;17(9):e1003246. doi: 10.1371/journal.pmed.1003246 (PMC7489547; doi:10.1371/journal.pmed.1003246)
Supplement: S4 Text — (DOCX) [file pmed.1003246.s005.docx]

**S4 Text. Example of a co-created video presentation.**

<https://www.youtube.com/watch?v=zir3Q_bqCsk>
